# RNA-seq profiling reveals differentially expressed genes as potential markers for vital reaction in skin contusion: a pilot study

**DOI:** 10.1080/20961790.2017.1349639

**Published:** 2017-07-18

**Authors:** Jingtao Xu, Rui Zhao, Ye Xue, Huanqin Xiao, Yanliang Sheng, Dong Zhao, Jietao He, Hongyan Huang, Qi Wang, Huijun Wang

**Affiliations:** a Department of Forensic Pathology, School of Forensic Mecicine, Southern Medical University, Guangzhou, China; b Department of Forensic Pathology, China Medical University School of Forensic Medicine, Shenyang, China; c Department of Pathology, The Third Affiliated Hospital, Sun Yat-Sen University, Guangzhou, China; d Department of Forensic Medicine, School of Basic Medicine, Jiamusi University, Jiamusi, China; e CollaborativeInnovation Center of Judicial Civilization, China and Key Laboratory of Evidence Science, China University of Political Science and Law, Ministry of Education, Beijing, China

**Keywords:** Forensic pathology, skin contusion, vital reaction, RNA-seq, differentially expressed genes

## Abstract

Detection of the vitality of wounds is essential in forensic practice. The present study used Illumina RNA-seq technology to determine gene expression profiles in contused mouse skin. In obtained high quality sequencing reads, the reads were mapped onto a reference transcriptome (Mus_musculus.GRCm38.83). The results revealed that there were 659 up-regulated and 996 down-regulated differentially expressed genes (DEGs) in contused mouse skin. The DEGs were further analyzed using the Gene Ontology and the Kyoto Encyclopedia of Genes and Genomes databases. Genes from different functional categories and signalling pathways were enriched, including the immune system process, immune response, defense response, cytokine–cytokine receptor interaction, complement and coagulation cascades and chemokine signalling pathway. Expression patterns of 11 DEGs were verified by RT-qPCR in mice skins. In addition, alterations of five DEGs were also analyzed in postmortem human wound samples. The results were in concordance with the results of RNA-seq. These findings suggest that RNA-seq is a powerful tool to reveal DEGs as potential markers for vital reaction in terms of forensic practices.

## Introduction

In forensic practice, detection of the vitality of wounds is essential to clarify the causal relationship between the cause of death and wounds detected in cadavers. However, it is sometimes difficult to discriminate antemortem wounds from postmortem damages in forensic practices, because of the lack of macroscopic objective evidences, especially when they are inflicted very close to the time of death [1]. Previous studies indicated that several functional categories and signalling pathways were useful for the determination of wound vitality and wound age estimation [1–7].

RNA-seq is a technique for RNA profiling based on the next-generation sequencing, which is a highly sensitive and accurate tool for measuring expression across the transcriptome. It can provide an expression profile with a greater and reproducible dynamic range. In recent years, the RNA-seq technique has been successfully applied to many research projects [8].

The present study used Illumina RNA-seq technology to determine gene expression profiles in contused mouse skin and discussed the availability of using this technique to reveal differentially expressed genes (DEGs) as potential markers for vital reaction.

## Materials and methods

### Ethical statement

This study was reviewed and approved by the ethics committee of Southern Medical University Institutional Board (Guangzhou, China). All sampling methods were carried out in accordance with the regulations of the Methods of extraction, fixation, packing and inspection of forensic pathology of The People's Republic of China Public Safety Industry Standard (GA/T 148-1996) and Forensic pathology materials extraction, fixed operating instructions of Southern Medical University (NYSJ-JS-BL04). No specific ethical application/decision number is needed for this particular study.

### Animal experimental protocol

In total, 16 male BALB/c mice (eight mice for contusion, eight mice for control, 7–9 weeks old; (25 ± 3) g) were obtained from the Laboratory Animal Centre of Southern Medical University. All animals were anesthetized with ether inhalation, and then shaved to expose skin area with an electric razor. Dorsal skin was picked up at the midline, and two layers of skin were compressed by a flat-mouth tong for 10 s (9 mm × 9 mm, SHE.K, China). This procedure generated two full-thickness contusions with one on each side of the midline. After 30 min, the mice were sacrificed under deep anesthesia with pentobarbital sodium (60 mg/kg i.p.). A 5 mm × 5 mm contused skin was excised from the centre of the left contused skin where subcutaneous bleeding can be observed. Control skins were collected from non-contused mice. Furthermore, abdominal skin samples from the injury animals were also collected for further examination. Skin samples were immediately submerged in 1 mL of RNA stabilization solution (RNAlaterTM, Ambion, Austin). During preparation of the samples, to reduce the influence of individual variations and also to save cost and time, the skins of four mice from each group were pooled to obtain one RNA sample, in which the total RNA was isolated by using TRIzol reagent (Invitrogen, Carlsbad, CA, USA) according to the manufacturer's protocol. Two RNA samples from each group were prepared and designated as Ctrl1, Ctrl2, Injury1 and Injury2.

### RNA sequencing and bioinformatics analysis

After generating RNA-seq cDNA libraries, RNA-seq was performed on Illumina Hiseq 2500. The Trim Galore method was used to dynamically remove the poor-quality segments and to connect the sequence segments. FastQC method was adopted for quality control analysis. Raw reads for each sample were mapped to the mouse reference genome (Mus_musculus.GRCm38.83) with STAR method [9]. The expression levels of the transcripts were calculated by fragments per kilobase of transcript per million fragments mapped (FPKM) values. DEGs were defined as fold change >2 times (absolute value of Log_2_ Ratio >1) based on their FPKM values between the groups, which were identified using DESeq2 method.

The DEGs (control group *vs*. injury group) were selected for gene ontology (GO), Kyoto Encyclopedia of Genes and Genomes (KEGG) pathways and Protein–Protein Interactions (PPI) analysis, by using the DAVID (https://david.ncifcrf.gov) [10] and the STRING database (http://string-db.org) [11]. The GO project is an international standardized gene functional classification system which describes the gene attributes, including the biological process (BP), molecular function (MF) and cellular component (CC). The KEGG database is the major public pathway-related database. The STRING database can provide a critical assessment and integration of PPI, including direct (physical) as well as indirect (functional) associations.

### Autopsy samples

Human skin wound samples of forensic autopsy cases (*n* = 6) at our institute were examined. A 5 mm × 5 mm contused skin was excised from the centre of the injured skin of fatal wound where bruises and subcutaneous bleeding can be observed. The cases comprised three males and three females, between 26 and 52 years of age. All deaths were due to traumatic injuries resulting from traffic accidents. There is only one wound per subject analyzed. Meanwhile, intact regions without any injury were taken from the abdominal skins of the same individuals as control. The demographics of the study subjects are described in Table 1. Postmortem interval is defined as the estimated time from death to autopsy, warm time is the time between death and cold storage of the body and survival time is the estimated period from the onset of the fatal insult to death; these were estimated on the basis of autopsy findings and circumstantial evidence recorded in autopsy documents.

**Table 1.** Case profiles.

Case	Age (years)	Gender	Cause of death	Survival time (min)	PMI (h)	Warm time (h)	Injured site	Injured type
1	30	M	Traumatic injury	20	39	16	Brain	Abrasion and contusion
2	26	M	Traumatic injury	40	28	12	Abdomen	Contusion
3	45	M	Traumatic injury	30	46	22	Chest	Abrasion and contusion
4	41	F	Traumatic injury	40	40	18	Abdomen	Contusion
5	32	F	Traumatic injury	10	32	13	Chest	Contusion
6	52	F	Traumatic injury	20	28	9	Brain	Abrasion and contusion

M: male; F: female; PMI: estimated postmortem interval.

### Quantitative real-time reverse transcriptase polymerase chain reaction (RT-qPCR)

Briefly, cDNA copies of total RNA were obtained using PrimerScript RT reagent Kit (TaKaRa, Japan). RT-qPCR reactions were run in 48-well reaction plates with an Illumina Eco Real-Time PCR System and a SYBR green kit (TaKaRa, Japan), according to the manufacturer's recommendations. GAPDH (glyceraldehyde-3-phosphate dehydrogenase) was used as an endogenous control for the RT-qPCR, and the relative expression levels were determined by the 2^−^^△△Ct^ method [12]. The RT-qPCR conditions, thermal cycler parameters and gene-specific primers used for amplification are listed in Supplemental Table S1.

## Statistic

All the RT-qPCR experiments were performed in triplicate, and the results were reported as the mean ± SEM. Correlation analyses between RNA-seq and RT-qPCR data were performed using linear regression (Pearson correlation analysis). The non-parametric Mann–Whitney *U*-test was used for comparisons between individual groups. Statistical analyses were performed using GraphPad Prism version 5.01 (GraphPad Software, San Diego, USA). Values of *P* < 0.05 were considered as statistically significant.

## Results

### Summary of the raw sequence reads

In total, 31.01 million, 30.16 million, 24.18 million and 21.47 million clean reads were obtained from Ctrl1, Ctrl2, Injury1 and Injury2, respectively (Table 2), of which 85.55% (Ctrl1), 86.19% (Ctrl2), 78.86% (Injury1) and 83.00% (Injury2) were uniquely aligned to the mouse reference genome (Mus_musculus.GRCm38.83).

**Table 2.** Summary of sequencing and mapping results.

Sample	Total reads	Total mapped	Total mapped ratio (%)	Unique mapped	Unique mapped ratio (%)
Ctrl1	31 005 734	28 207 321	90.97	26 525 229	85.55
Ctrl2	30 158 418	27 387 573	90.81	25 993 426	86.19
Injury1	24 184 524	21 546 034	89.09	19 071 896	78.86
Injury2	21 467 012	19 364 236	90.20	17 818 492	83.00

### Differential expression genes

Using DESeq2 method, a total of 659 up-regulated and 996 down-regulated DEGs among 26 962 genes were detected in contused mouse skin. A complete list of the differentially expressed transcripts is included in Supplementary Table S2.

### The RNA-seq results were verified by RT-qPCR

**Figure 1.**
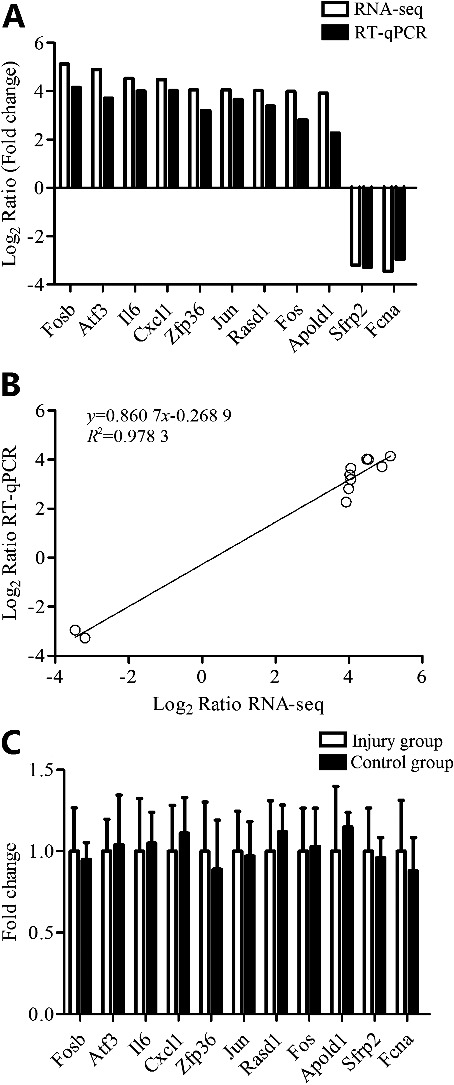
RT-qPCR validation of 11 DEGs. (A) The transcript expression fold changes measured by RNA-seq and RT-qPCR are indicated by white and black columns, respectively. (A and B) The expression levels of nine up-regulated genes, Fosb, Atf3, IL6, Cxcl1, Zfp36, Jun, Rasd1, Fos and Apold1, two down-regulated genes, Sfrp2 and Fcna, were all consistent with the RNA-seq data. (B) The regression equation between RNA-seq (*x*) and RT-qPCR (*y*) data (Log_2_ Ratio) is *y* = 0.860 7*x* − 0.268 9, while the *R*^2^ is 0.978 3. (C) Those 11 DEGs showed no difference between abdominal skins of the injury group (RT-qPCR-A) and control group (RT-qPCR-B).

All qPCR reactions yielded a single peak on the dissociation curve, indicating specific amplifications achieved with those primers. To evaluate the RNA-seq results, 11 DEGs (nine up-regulated genes, Fosb, Atf3, IL6, Cxcl1, Zfp36, Jun, Rasd1, Fos and Apold1; two down-regulated genes, Sfrp2 and Fcna) were selected randomly for RT-qPCR confirmation in mice skins [13]. For these genes, the results of RT-qPCR were consistent with the RNA-seq data (Figure 1(A,B)). The regression equation between RNA-seq (*x*) and RT-qPCR (*y*) data (Log_2_ Ratio) is *y* = 0.860 7*x* − 0.268 9, while the *R*^2^ is 0.978 3, which confirmed the decent RNA-seq data obtained in the present study for sound conclusions.

Furthermore, abdominal skin samples from the injury animals were also examined. The above-mentioned 11 DEGs showed no difference between abdominal skins of the injury group and control group (Figure 1(C)).

### GO and KEGG analyses of differentially expressed RNAs

**Figure 2.**
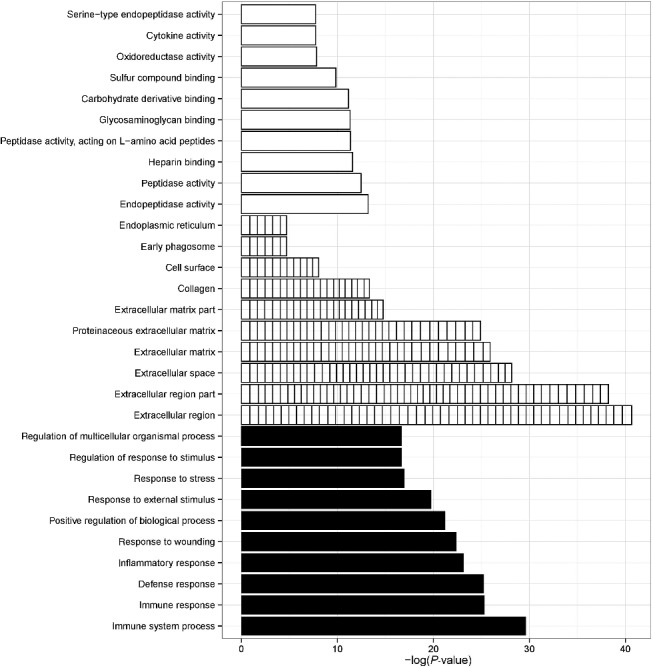
Gene ontology analysis. The GO analysis revealed that the “immune system process”, “endopeptidase activity” and “extracellular region” were the enriched GO terms containing DEGs belonging to BP (white columns), MF (fence columns) and CC (black columns), respectively.

**Figure 3.**
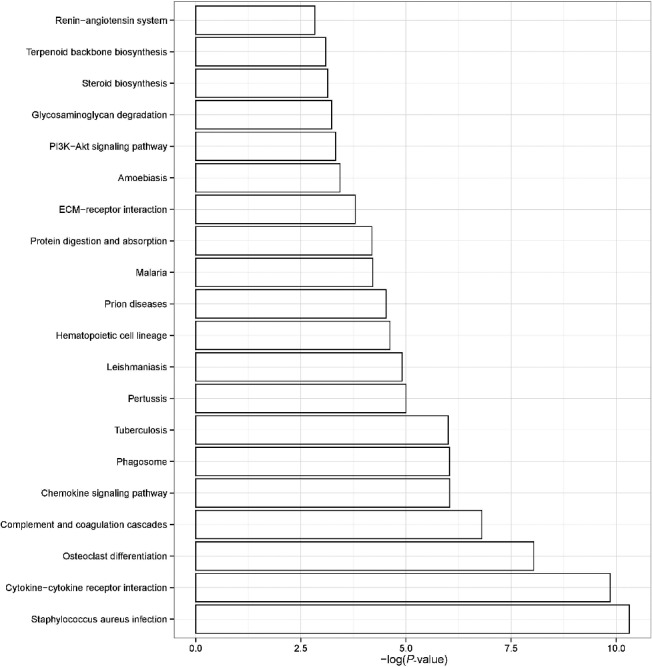
KEGG pathway enrichment analysis. The KEGG pathway analysis demonstrated that the “Staphylococcus aureus infection”, “Cytokine−cytokine receptor interaction” and “Osteoclast differentiation” were the most enriched pathways containing DEGs. The enrichment factors of the pathway are shown on the x-axis.

**Figure 4.**
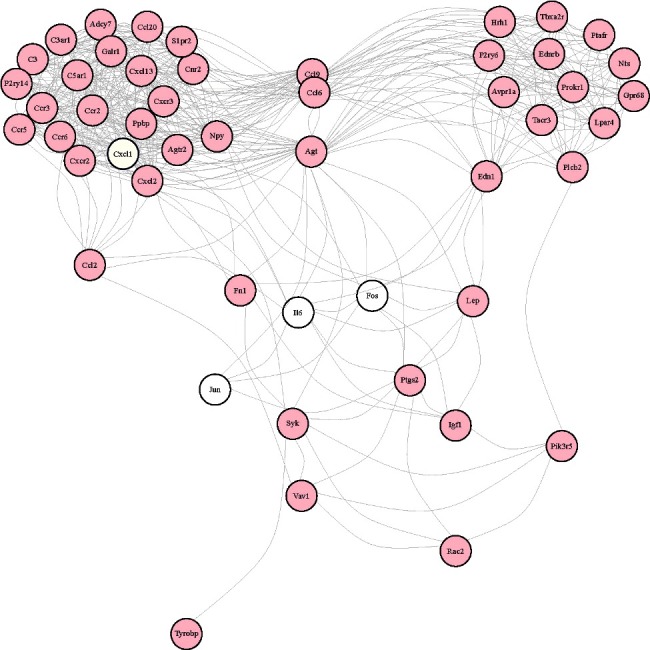
PPI analysis. Direct (physical) and indirect (functional) associations by PPI analysis.

GO categories were used to describe the BP, MF and CC of the DEGs between the injury group and control group. The GO analysis revealed that the “immune system process”, “endopeptidase activity” and “extracellular region” were the enriched GO terms containing the DEGs belonging to BP, MF and CC, respectively (Figure 2). The KEGG pathway analysis demonstrated that the “Staphylococcus aureus infection”, “Cytokine–cytokine receptor interaction” and “Osteoclast differentiation” were the most enriched pathways containing DEGs (Figure 3). The STRING software was used to analyze PPI networks of the DEGs (Figure 4). The results of GO, KEGG analysis and PPI networks of the up/down-regulated DEGs are shown in Supplementary Figures S3–8.

### RT-qPCR results in postmortem human skin wounds

**Figure 5.**
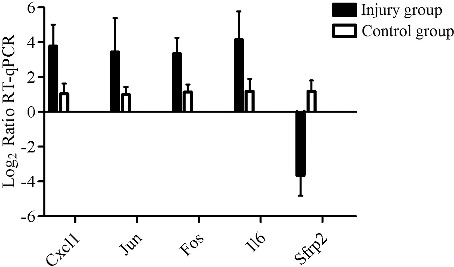
RT-qPCR results in postmortem human skin wounds. In line with animal experiments, Cxcl1, Jun, Fos and IL6 RNA expression levels were significantly up-regulated in postmortem human skin wounds than in intact skins, while Sfrp2 was down-regulated.

In the wounded and the intact human skin tissues from the same individual, five DEGs (four up-regulated genes, Cxcl1, Jun, Fos and IL6; one down-regulated gene, Sfrp2) were randomly selected for RT-qPCR confirmation. In line with animal experiments, Cxcl1, Jun, Fos and IL6 RNA expression levels were significantly up-regulated in postmortem human skin wounds than in intact skins, while Sfrp2 was down-regulated. Briefly, for all tested genes, the RNA expression levels analyzed by RT-qPCR in postmortem human skin wounds were similar to the results of the RNA-seq (Figures 1(A) and 5). Furthermore, there were no gender-related differences, or age or survival time or postmortem interval dependence in mRNA levels of those five DEGs on Pearson correlation analysis (*R*^2^ < 0.1, *P* > 0.05).

## Discussion

The present study, for the first time, used Illumina RNA-seq technology to determine the gene expression profiles in contused mouse skin. Millions of clean reads were obtained from contused and control skin samples, which were uniquely aligned to the mouse reference genome (Mus_musculus.GRCm38.83). The mapping percentage was pretty comparable, ensuring reliable downstream analysis.

A total of 659 up-regulated and 996 down-regulated DEGs were detected in contused mouse skin. The GO analysis revealed that the “immune system process”, “endopeptidase activity” and “extracellular region” were the enriched GO terms containing the DEGs belonging to BP, MF and CC, respectively. The KEGG pathway analysis demonstrated that the “Staphylococcus aureus infection”, “Cytokine–cytokine receptor interaction” and “Osteoclast differentiation” were the most enriched pathways. These bioinformatic analyses might help us to better understand the process of wound healing, especially in the early phase.

In addition, 11 DEGs were selected randomly and their expression levels were examined using RT-qPCR. Those 11 DEGs showed no difference between abdominal skins of the injury group and control group. Furthermore, the expression levels of these genes in injury and control groups were all consistent with the RNA-seq data, which confirmed the decent RNA-seq data obtained in the current study for reliable conclusions. In order to evaluate forensic availability of those DEGs, gene expressions of five DEGs were detected in postmortem wound samples. In line with animal experiments, Cxcl1, Jun, Fos and IL6 RNA expression levels were significantly up-regulated in postmortem human skin wounds than in intact skins, while Sfrp2 was down-regulated (the complete list of the DEGs is included in Supplementary Table S2).

Schober reported that Fosb and IL6 were increased in the cerebellum of acute deaths due to traumatic brain injury [14]. IL1 beta is a pro-inflammatory cytokine that has been highlighted early after injury. Bai reported an IL1 beta RNA overexpression occurring approximately 30 min after injury [15]. Takamiya and colleagues also demonstrated that IL6 showed exclusive expressions in the process of dermal wound healing [16]. The up-regulated expression of ILs may indicate the important role in the early phase of an inflammatory cascade. Michael and colleagues found that transcription factors (Fos, Jun) were up-regulated in the pericontusional zone of traumatic brain injury (TBI) patients [17]. Fos and JUN have been defined as the canonical genes of the early response pathway and were similarly elevated in the present study. According to Kameyama'study, one day after the dorsal skin incisions, wounds on male Sprague-Dawley rats showed the statistically significant increase in the RNA expressions for CXCL2 [18]. CXCL2 is a neutrophil chemokine that encodes secreted proteins involved in immunoregulatory and inflammatory processes. Our results showed up-regulation of Fosb, IL6, IL1 beta, Fos, JUN and CXCL2, which were in concordance with these studies, indicating immediate-early growth response, immunoregulatory and inflammatory process in the early stage of contusion, though the activation of these genes in different tissues seems to be diverse from each other. Sfrp2 is considered to be a Wnt antagonist that regulates the Wnt signalling pathway [19]. Several studies suggest a decrease of Sfrp2 in a variety of cancers; further evidences indicate that Sfrp2 is able to inhibit the Wnt-induced increase in the levels of free β-catenin and influence inflammation and tumour cell proliferation [20,21]. In the present study, Sfrp2 is down-regulated in the contused skin, indicating an activation of Wnt pathway-mediated inflammation. Our results are in concordance with these studies.

However, parts of our results are not consistent with previous studies. Ohshima showed that IL10 mRNA was a possible indicator of wound vitality. Fifteen minutes after incision, a rapid increase in IL10 mRNA was detected [22]. The mRNA expression levels of IL10, IFN γ and TNF α have been reported up-regulated in previous study [16]. However, in our study, those markers showed no difference between the injury group and control group. These inconsistent results may be caused by the use of different models of injury. Several groups used different wound models to determine the wound vitality and estimate the wound age [1–7]. The advantage with the cut wound model is that a large number of injured vessels along the cut margin can be obtained. However, contusions with differently distributed injured vessels can usually be found in forensic practice. Therefore, our pilot study is aimed at providing DEGs as potential markers for vital reaction in skin contusion. The DEGs from animal wound models cannot be directly used in human samples without careful validation. In addition, only five DEGs were examined in postmortem human samples in the present study. Further studies with more human samples and various types of wound samples are needed to confirm the DEGs as potential markers for vital reaction.

In addition, because of the differences in translation efficiency or RNA/protein kinetics, and the lower sensitivity of immunostaining in detecting changes in gene products than that with quantitative analyses of gene expressions using RT-qPCR, in the present study, only RNA levels of DEGs were analyzed. Further investigation, using some semi-quantitative methods such as ELISA or Western blot, is needed to detect the protein levels, and to compare the protein levels with RNA levels.

Collectively, this pilot study used Illumina RNA-seq technology to determine gene expression profiles in contused mouse skin and verified some DEGs in postmortem wound samples. Our findings indicate that RNA-seq is a powerful tool to reveal DEGs as potential markers for vital reaction, though further investigations are required to confirm these results.

## Supplementary Material

supp_mat_TFSR_1349639.zip
