# Supplementary figures and images for: RNA-seq profiling reveals differentially expressed genes as potential markers for vital reaction in skin contusion: a pilot study
Source: Forensic Sci Res. 2017 Jul 18;3(2):153–60. doi: 10.1080/20961790.2017.1349639 (PMC6197083; doi:10.1080/20961790.2017.1349639)

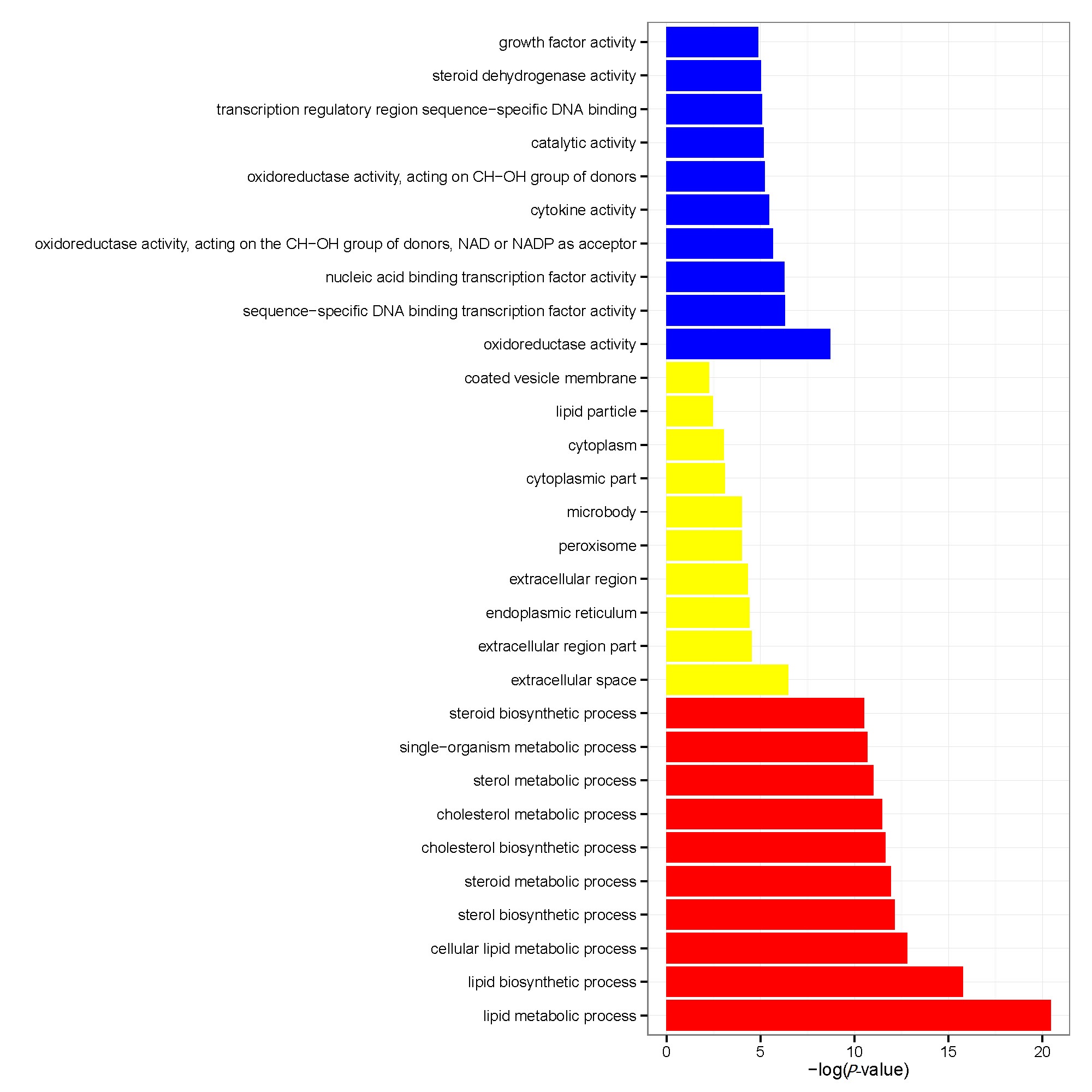

Supplement: supp_mat_TFSR_1349639.zip [file TFSR_A_1349639_SM2505.zip › supp_mat_TFSR_1349639/Supplementary Material 3.jpg]

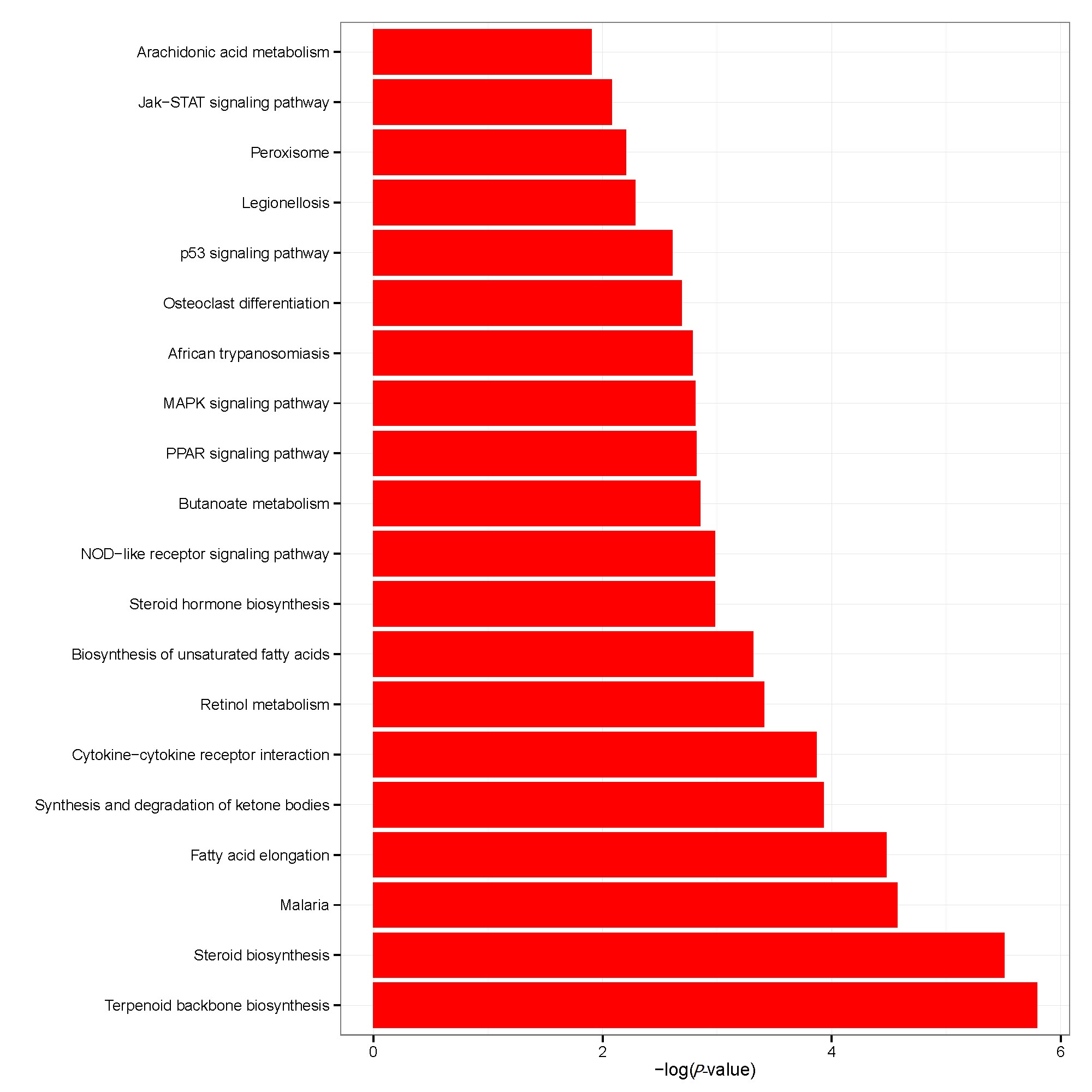

Supplement: supp_mat_TFSR_1349639.zip [file TFSR_A_1349639_SM2505.zip › supp_mat_TFSR_1349639/Supplementary Material 4.jpg]

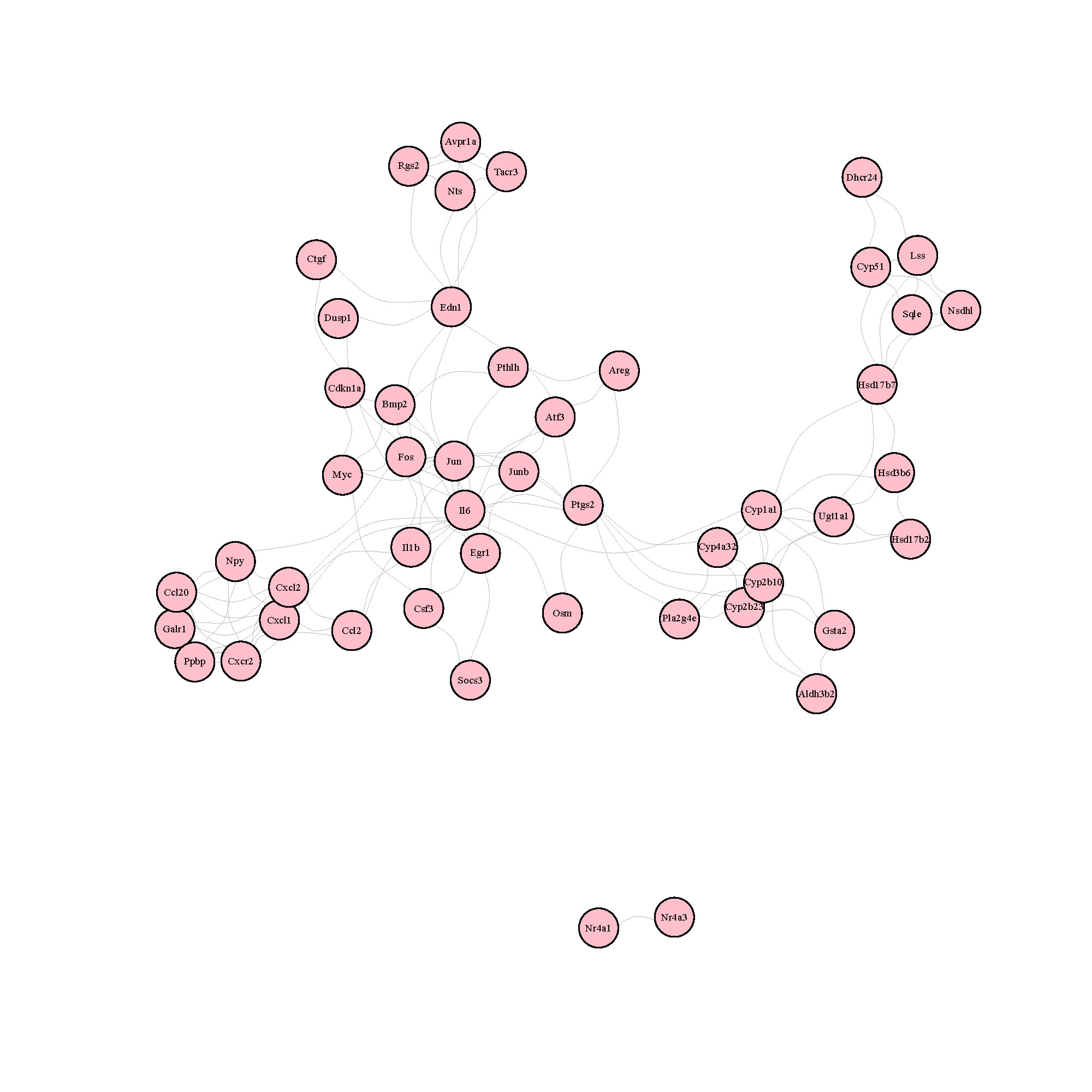

Supplement: supp_mat_TFSR_1349639.zip [file TFSR_A_1349639_SM2505.zip › supp_mat_TFSR_1349639/Supplementary Material 5.jpg]

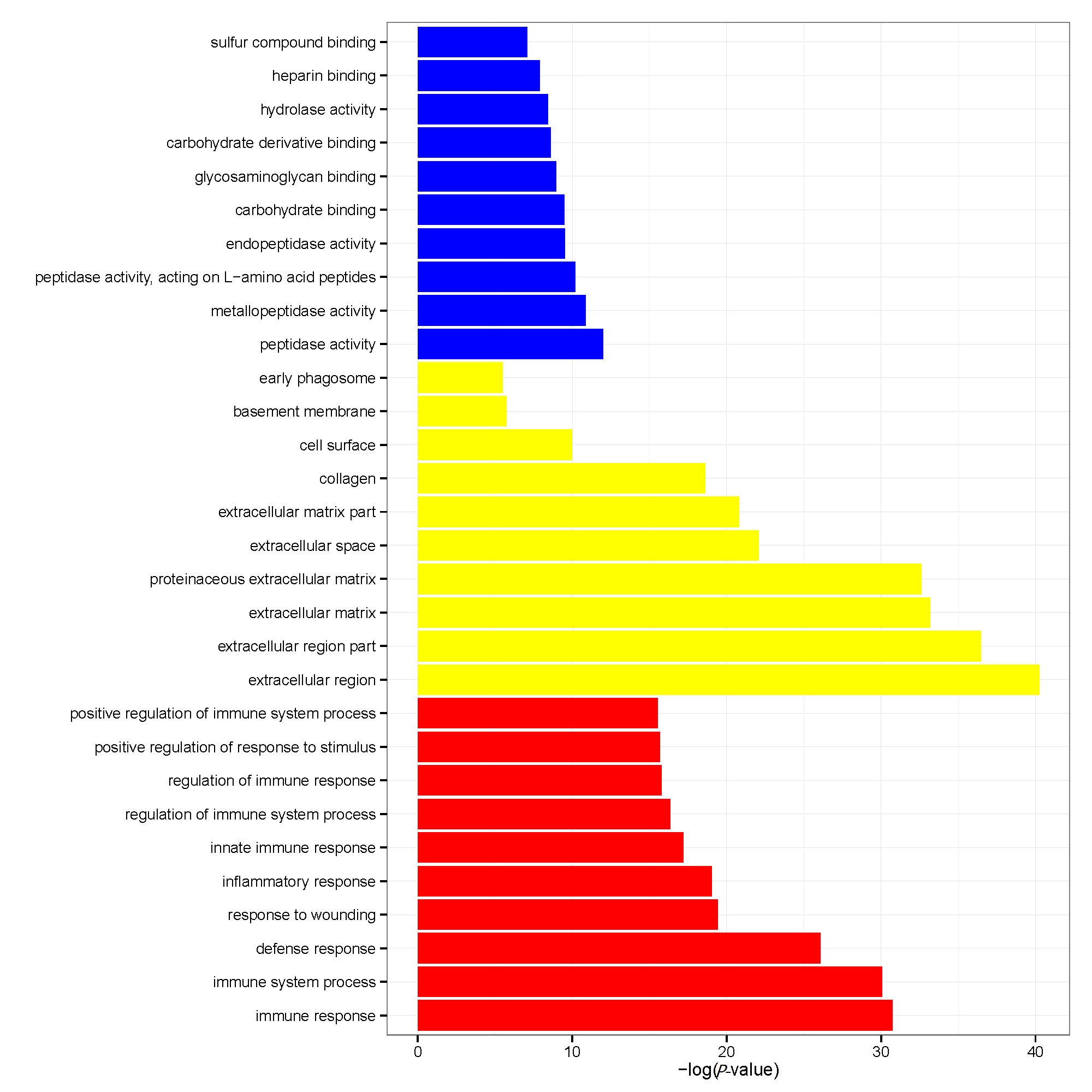

Supplement: supp_mat_TFSR_1349639.zip [file TFSR_A_1349639_SM2505.zip › supp_mat_TFSR_1349639/Supplementary Material 6.jpg]

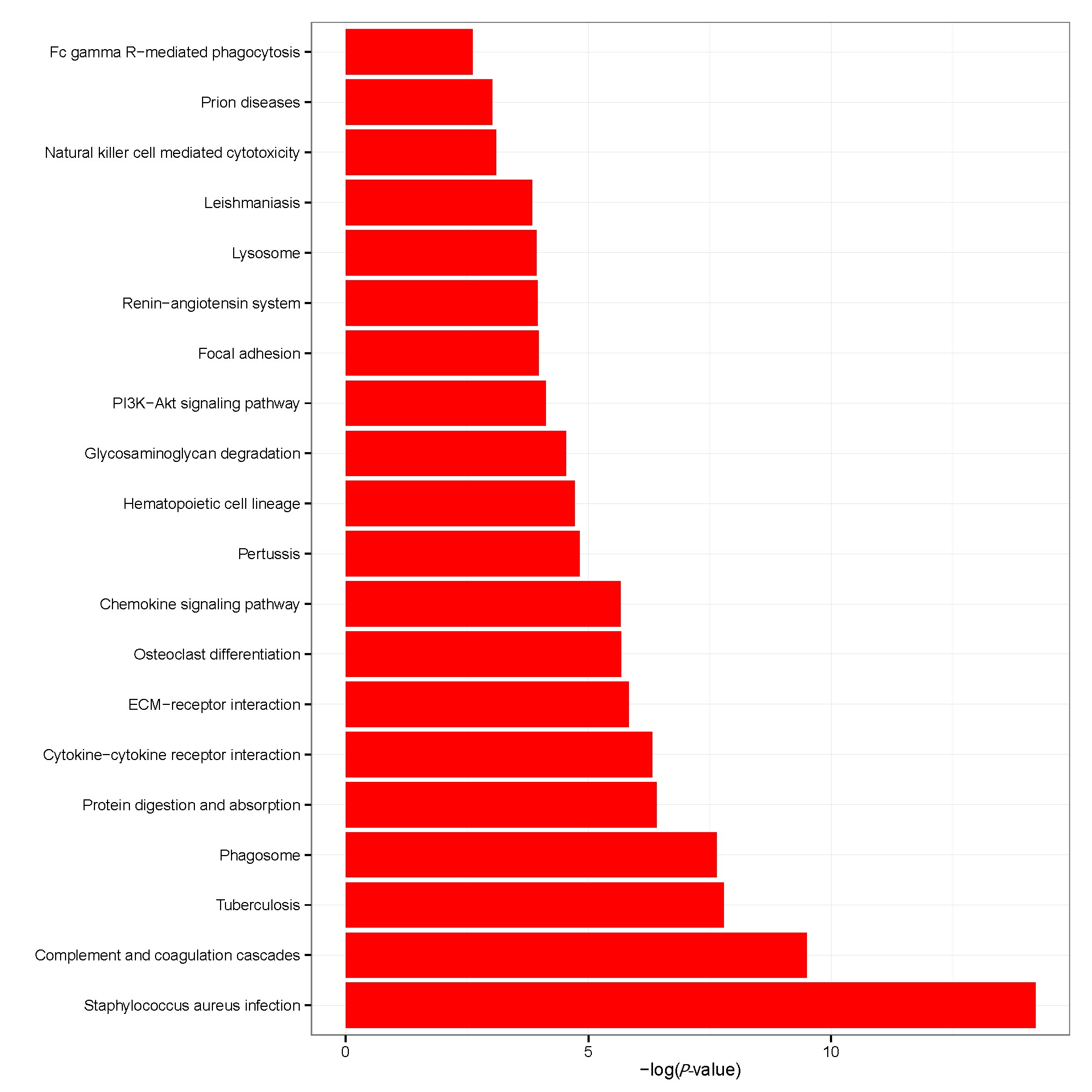

Supplement: supp_mat_TFSR_1349639.zip [file TFSR_A_1349639_SM2505.zip › supp_mat_TFSR_1349639/Supplementary Material 7.jpg]

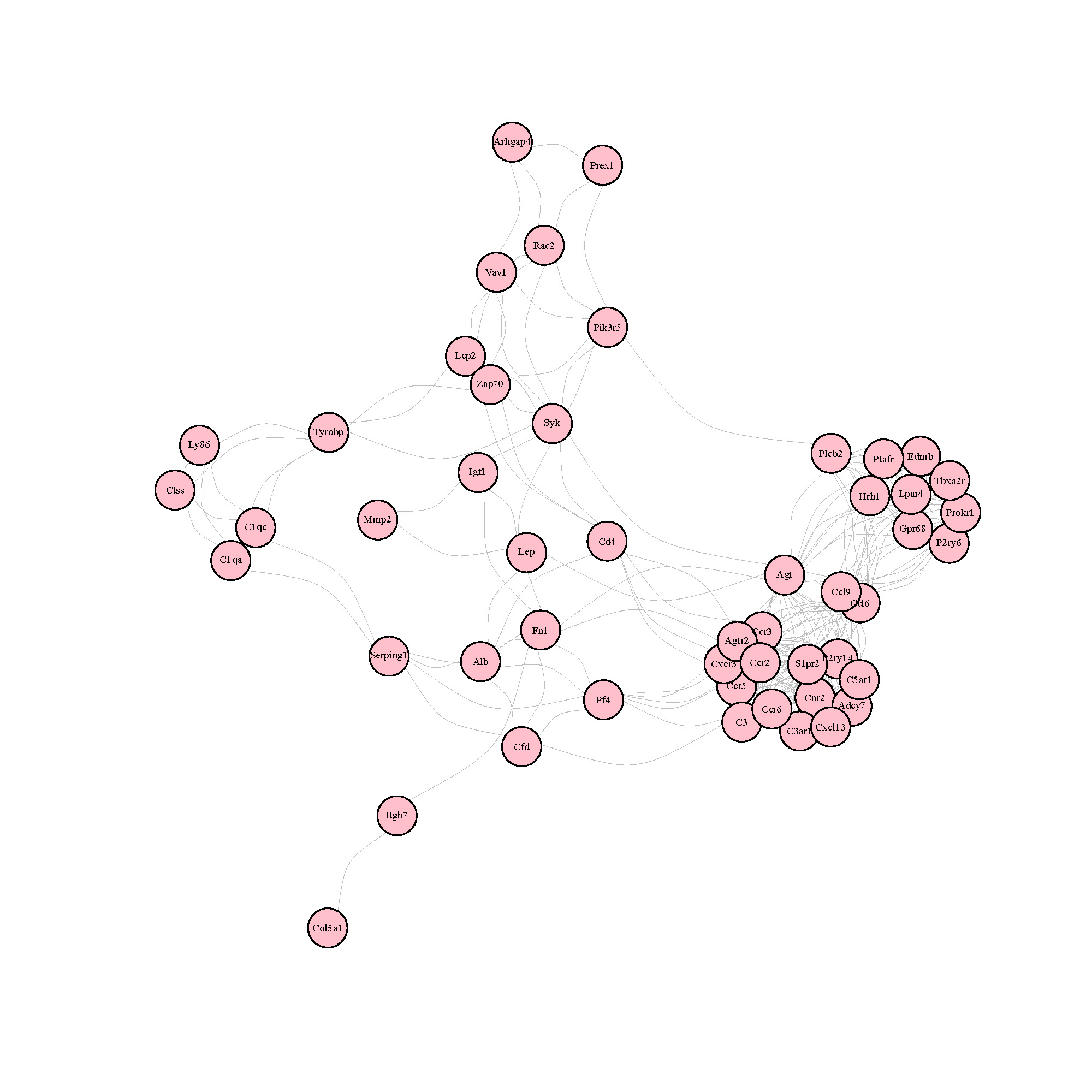

Supplement: supp_mat_TFSR_1349639.zip [file TFSR_A_1349639_SM2505.zip › supp_mat_TFSR_1349639/Supplementary Material 8.jpg]
